# The use of radiofrequency in cancer

**DOI:** 10.1038/sj.bjc.6602582

**Published:** 2005-05-03

**Authors:** A R Gillams

**Affiliations:** 1 Department of Medical Imaging, The Middlesex Hospital, Mortimer Street, London W1T 3AA, UK

**Keywords:** thermal ablation, radiofrequency, hepatocellular carcinoma, liver metastases, inoperable pulmonary malignancy, renal cell carcinoma

## Abstract

Radiofrequency ablation (RFA) provides an effective technique for minimally invasive tissue destruction. An alternating current delivered via a needle electrode causes localised ionic agitation and frictional heating of the tissue around the needle. Image-guided, percutaneous ablation techniques have been developed in most parts of the body, but the most widely accepted applications are for the treatment of hepatocellular carcinoma (HCC) in early cirrhosis, limited but inoperable colorectal liver metastases, inoperable renal cell carcinoma and inoperable primary or secondary lung tumours. The procedures are well tolerated and the complication rates low. Patients with coexistent morbidity who are not suitable for surgery are often able to undergo RFA. Most treatments in the lung, kidney and for HCC are performed under conscious sedation with an overnight hospital stay or as a day-case. Larger more complicated ablations, for example, in hepatic metastases may require general anaesthesia. Limitations of RFA include the volume of tissue that can be ablated in a timely fashion, that is, most centres will treat 3–5 tumours up to 4–5 cms in diameter. Early series reporting technical success and complications are available for lung and renal ablation. Liver ablation is better established and 5-year survival figures are available from several centres. In patients with limited but inoperable colorectal metastases, the 5-year survival ranges from 26 to 30% and for HCC it is just under 50%. In summary, RFA provides the opportunity for localised tissue destruction of limited volumes of tumour; it can be offered to nonsurgical candidates and used in conjunction with systemic therapy.

Image guided ablation dates to 1989 when the first US guided interstitial laser treatments were performed in liver metastases and the first percutaneous ethanol injections were reported for the treatment of small hepatocellular carcinoma (HCC). Rapid technological developments have resulted in the development of a number of ablative techniques, higher power generators and a range of different electrode designs. Current designs permit ablation of much larger volumes of tissue than was originally possible. Radiofrequency ablation (RFA) has been used in a wide range of locations and applications including adrenal metastases, primary colorectal cancer recurrence and malignant pelvic or para-aortic lymphadenopathy (Lees and Gillams, 2005), but in this article we will consider the main areas where RFA has proven effective or is likely to do so.

## TECHNICAL FEATURES

Radiofrequency ablation uses needle electrodes to deliver an alternating current that generates ionic agitation, localised frictional tissue heating and cell death. A range of electrode designs is available ranging in caliber from 14 to 1 7 G. Most are designed to be inserted percutaneously using image guidance. Various design features have been put forward (water cooling, saline perfusion, monopolar or bipolar, coil or expandable designs), all aimed at increasing the volume of confluent necrosis that can be achieved per unit time. Ultrasound (US) is the preferred technique for electrode placement but where US is not possible, for example, in lung ablation or in US occult liver tumours, then CT or MR are used. Assessment of the efficacy of ablation is achieved using contrast-enhanced CT or MR. Absent enhancement denotes the area of necrosis. Thermal techniques result in coagulation necrosis and an ‘instantaneous thermal fixation’ effect. Tissue architecture is preserved, routine histological stains are misleading and enzymatic assays are required to establish tissue nonviability (Ni *et al*, 2005).

## LIVER METASTASES

### Colorectal

This patient population represents one of the most common indications for RFA in the UK. Liver resection is currently considered the first line of treatment for those with surgical disease, but there are many patients who are not suitable for resection either because of comorbidity, tumour location or inadequate hepatic reserve (most commonly in patients who have undergone a previous resection). These patients may well benefit from RFA to the liver either in isolation or in conjunction with systemic chemotherapy. As liver metastases usually arise in an otherwise normal liver, the extent of disease that can be safely treated is greater than for patients with HCC arising on a background of cirrhosis. Most centres will accept patients with as many as five tumours, maximum diameter 5 cm. We have published our survival results in 167 patients with colorectal metastases treated with RFA but have now treated over 215 patients (Gillams and Lees, 2004). A total of 96 patients conformed to the above criteria and their survival was a median, 1, 3 and 5 years from the diagnosis of liver metastases of 38 months, 99, 59 and 29% and from the time of first thermal ablation was 31 months, 89, 33 and 20%. Other groups have reported similar survival results and the Milan group reported their results in 117 patients with up to four metastases, mean diameter 2.8 cm. The vast majority (103 out of 117 (88%)) of their patients had either one or two tumors. The median survival in this cohort, who had more limited disease than our cohort, was 36 months and their 3-year survival was 46% (Solbiati *et al*, 2001). Vogl *et al* (2004) have reported a similar median survival of 35 months in patients with five tumours or less, maximum diameter 5 cm. This group uses a different thermal technique (neodymium yttrium aluminium garnet (NdYAG) laser ablation) and a combination of CT guidance for applicator placement and high field MR monitoring of the thermal effect.

**Figure 1:**
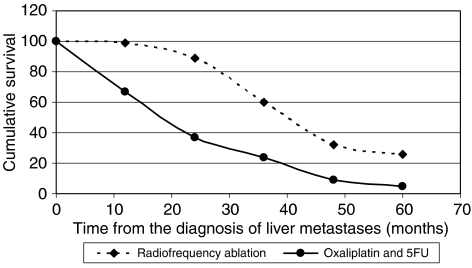
A theoretical projection of survival following RFA (in patients with CLOCC acceptance criteria from our database) *vs* published Oxaliplatin and 5FU chemotherapy results.

Analysis of patients with small solitary lesions, that is, surgical disease but in those who were not suitable for surgery, reveals even better survival figures. In our cohort, 34 patients had small solitary tumours with a median diameter of 2.5 cm, the mean and 3-year survival from ablation was 67 months and 66%, respectively. Two retrospective comparisons between surgery and RFA suggest comparable survival between the two modalities (Elias *et al*, 2002; Oshowo *et al*, 2003); however, no prospective studies are underway. A different approach is to use RFA as a ‘test of time’ – this has been explored by the Milan group who have offered RFA to surgical candidates (Livraghi *et al*, 2003a). Those who do not develop further disease are spared a resection, those who develop more extensive disease such that they are no longer operable on short-term follow-up are spared an un-necessary and ineffective surgical resection and those that recur can undergo resection. In their cohort of 88 patients, 60% achieved complete ablation and did not require resection. An on-going trial, sponsored by The European Organisation for Research and Treatment of Cancer (EORTC), is looking at a different patient population, those with inoperable colorectal liver metastases. This trial aims to compare the effect of ablation in conjunction with systemic chemotherapy with systemic chemotherapy alone, that is, Chemotherapy + Local ablation *vs* Chemotherapy (CLOCC) trial. The acceptance criteria are more generous than traditional acceptance criteria. Patients can have as many as nine metastases with a maximum diameter of 4 cm. We have retrospectively analysed our data according to the CLOCC acceptance criteria. A theoretical projection comparing our results with published Oxaliplatin and 5 fluorouracil (5FU) data is shown in Figure 1 (Giacchetti *et al*, 2000). This projection would suggest a useful survival advantage for the addition of ablation to systemic chemotherapy but only the on-going prospective study will provide the required evidence.

Complication rates of RFA are low with a mortality of <0.8% and morbidity of 5–10%. Collateral damage can largely be avoided by isolating the liver from adjacent vulnerable structures, for example, colon or duodenum. This is usually readily achievable by the instillation of < 1 l of 5% dextrose through a 19 G spinal needle positioned between the ablation area and the vulnerable viscus. Major bile ducts are susceptible to thermal injury and need to be cooled. Bleeding is uncommon in patients with normal coagulation and a normal liver parenchyma. Although one centre has reported very high rates of tumour seeding following biopsy and RFA (Llovet *et al*, 2001), in all other centres tumour seeding is exceptional – 0.5% in one multicentre study which included 41 centres and over 2000 patients (Livraghi *et al*, 2003b). Secondary infection of an ablation zone can occur, usually as a late complication. Patients with bilio-enteric anastomoses or biliary stents are particularly likely to develop secondary infection presumably due to reflux of intestinal organisms into the biliary tree and therefore these patients should receive 3 months of rotating oral antibiotics post-ablation.

### Breast

The median survival for patients with metastatic breast cancer is 2 years; however, those with hepatic metastases have a worse prognosis. A small group of patients, 5–12%, have metastases confined to the liver. Some centres offer hepatic resection in this group and reported 3-year survival rates are 35–71%. The median survival of patients with secondary liver metastases following chemotherapy is 4.5 months; agents such as docetaxel may only improve survival to 9–14.7 months. Liver metastases tend to be resistant to hormonal manipulation, often being negative for hormone receptors. Liver resection is being considered more frequently but where resection is not possible RFA can be used for patients with limited liver metastases who either have no extra-hepatic disease or who have stable, controlled extra-hepatic disease. We have follow-up on 19 patients treated with RFA, eight had disease confined to the liver, with 11 having stable extra-hepatic disease in addition. Seven patients with disease confined to the liver at presentation are alive, as are six with extra-hepatic disease, median follow up after RFA was 15 months (range 0–77). Survival at 30 months was five out of 12 (41.6%), in addition seven followed up for a median of 14 months (range 2–29) remain alive and disease free. Livraghi *et al* treated 24 patients; 10 (41.6%) were disease free at a median follow up of 10 months. Therefore, there does appear to be a subgroup of patients who may benefit from local ablative therapy.

### Neuroendocrine

There are numerous treatment options for patients with neuroendocrine metastases, yet none is both widely applicable *and* effective in reducing tumour load. For many of these patients, the natural history of the disease whereby multiple often small metastases develop over a period of years renders them particularly suitable for a minimally invasive technique that can be repeated frequently while sacrificing the least amount of normal liver parenchyma. Radiofrequency ablation can be used to reduce hormone secretion and/or to control total tumour load. In our experience in 25 patients, we achieved local control of tumour volume in 14 out of 19 (74%) patients. There was a complete response in six, partial response in seven and stable disease in one at a median follow-up of 21 months (range 4–75). Relief or a reduction in hormone-related symptoms was achieved in nine of 14 (69%) with secreting tumours. The median survival from the diagnosis of liver metastases was 53 months (Gillams *et al*, 2005). Symptomatic carcinoid patients require peri-procedural IV Octreotide with careful monitoring during and after the procedure as ablation, like other interventional procedures, can result in hormone release and rapid changes in blood pressure.

## PRIMARY HEPATOCELLULAR CARCINOMA

Percutaneous, image-guided ablation has been used in the treatment of limited HCC for many years. Originally, chemical methods were used with either acetic acid or 100% ethanol. Ethanol is still used but increasingly thermal techniques such as radiofrequency, interstitial laser coagulation and microwave are becoming the preferred techniques. The presence of background cirrhosis in the vast majority of patients with HCC reduces the attractiveness of liver resection. Chemotherapy is ineffective and only recently met-analyses have suggested that chemoembolisation has an impact on survival (Camma *et al*, 2002). To be suitable for a local ablative technique, patients should have one HCC nodule less than 5 cm or as many as three nodules <3 cm in maximum diameter. The underlying liver disease should be no more advanced than Child Pugh class B. Retrospective comparisons of percutaneous ethanol injection (PEI) and resection show similar survival results (Livraghi *et al*, 1995). More recently, a prospective comparison of radiofrequency and PEI in 102 patients showed a significantly longer disease-free survival in the RFA group. The 1- and 2-year local recurrence-free survival rates were 98 and 96% in the RFA group compared to 83 and 62% in the ethanol injection group (*P*=0.002). There was a trend towards increased overall survival with RFA but this has not yet reached significance (Lencioni *et al*, 2003). Long-term survival post RFA has also been reported in 206 patients with hepatic cirrhosis and early-stage HCC. The overall survival was 97% at 1 year, 71% at 3 years and 48% at 5 years. Survival of Child-Pugh A patients (*n*=144; 76% at 3 years and 51% at 5 years) was significantly better than that of Child-Pugh B patients (*n*=43; 46% at 3 years and 31% at 5 years, *P*=0.0006). A subgroup of 116 patients with Child-Pugh class A cirrhosis and solitary HCC had 3- and 5-year survival rates of 89 and 61%, respectively (Lencioni *et al*, 2005). These results are sufficiently compelling and it seems likely that RFA will become the treatment of choice for early stage HCC.

## PULMONARY NEOPLASIA

Radiofrequency ablation can be equally well applied to small lung tumours, either primary or secondary. Several hundred treatments have been performed worldwide, a sufficient number to develop a reasonable safety profile (Steinke *et al*, 2004b). Overall, the complication rate is very similar to that seen following percutaneous lung biopsy. Pneumothorax rates are of the order of 40%, with only 10–15% requiring drainage or tube insertion. The need for intervention depends on the patient population and underlying lung disease, for example, in our cohort of >40 treatments in patients with metastatic pulmonary lesions, it has not yet been necessary to insert an intercostal drain. The pleural effusion rate is similar at 10%. The ideal RFA candidate is a small, peripherally located tumour. Again treatments can be performed under conscious sedation as an overnight or day-case procedure. CT guidance is required and CT fluoroscopy is useful. Successful ablation is denoted by the development of ground glass shadowing that completely encompasses the tumour. Initial consolidation should be larger than the original tumour and then shrink over time such that only a linear scar is visible at 12 months in 33% of successfully treated tumours (Steinke *et al*, 2004a). Another useful CT feature, which denotes successful ablation, is the absence of enhancement within the consolidated area. Most centres will treat patients with a limited number of metastases, for example, <3 per lung. Again the very focal effects of ablation favour small tumours <4 or 4.5 cm. Centrally placed tumours carry the additional risk of collateral damage and the increased likelihood that adjacent cooling blood vessels may protect tumour cells and result in an increased recurrence rate. Currently, RFA is offered to patients with small tumours who are not suitable for conventional treatment. Table 1 summarises the larger series in the literature. Most have concentrated on technical success and complications but Lee *et al* commented on survival. They reported on ablation of 32 tumours in 30 patients, 26 of whom had primary bronchogenic carcinoma. Complete ablation was achieved in all six tumours less than 3 cm in diameter and mean survival for this subgroup was 19.7 months *vs* 8.7 months for the remainder. The use of RFA in conjunction with radiotherapy and/or chemotherapy in the treatment of primary lung cancer is being explored (Jain *et al*, 2003). New emphasis on screening high-risk populations for the early detection of primary bronchogenic carcinoma will result in the detection of earlier cancers. Not all of these patients will be candidates for surgery, but may be candidates for RFA. As in the liver, RFA is also useful for the treatment of recurrence or new lesions in post surgical patients.

## RENAL CELL CARCINOMA

Nephron sparing techniques such as partial nephrectomy have been shown to be as effective as the traditional total nephrectomy, while providing a reduced morbidity and better preservation of renal function (Lee *et al*, 2000). Against this background, several groups have explored local ablation techniques in early small renal cell carcinoma and RFA has been very successful. Radiofrequency ablation is usually performed under conscious sedation, with a day-case or overnight stay, using US or CT guidance. Like HCC, the well-defined encapsulated lesions are more amenable to destruction by thermal techniques than ill-defined infiltrating tumours such as hepatic metastases. Most experience has been gained in patients who are not operable candidates or for whom nephron sparing is particularly critical, that is, those who have had previous contralateral nephrectomy, patients with multiple renal cell carcinomas notably those with von Hippel Lindau syndrome. Renal ablation is particularly successful for small, <4 cm, exophytic lesions. Central lesions are vulnerable to cooling from adjacent vessels resulting in incomplete ablation. In the largest series published to date, the success rate, that is, complete ablation on immediate contrast enhanced CT was 100% for exophytic tumours and 45% for central tumours (Lui *et al*, 2003). The complication rates are low and the collateral damage to intact nephrons minimised such that renal function remains stable.

Table 2 summarises the main papers published to date.

## SYMPTOMATIC PALLIATION

Occasionally, there is a role for RFA in symptom palliation. Radiofrequency ablation has been successfully applied to bony metastases to relieve pain. Charboneau and co-workers report marked reductions in pain and analgesic requirements following the treatment of limited, localised osteolytic bone metastases (Goetz *et al*, 2004). One caveat is that there should be a specific, clearly identifiable and localised area of bone destruction responsible for the patients' pain. Radiofrequency ablation has also been used to debulk tumours to reduce pressure symptoms, for example, dyspnoea or dysphagia.

## CONCLUSION

Radiofrequency ablation has proven effective as a local technique for tumour destruction. The complication profile is acceptable. The challenge now is to define in which patient populations and under what circumstances RFA should be used and how best to combine this local technique with systemic therapy.

## Figures and Tables

**Table 1:** Radiofrequency ablation in lung tumours

**Authors**	**No. of tumours/** **patients**	**Histology 1°/2°**	**Tumour size** **mean (range) or** **maximum cm**	**Complete ablation –** **no. of tumours**	**Length of** **follow-up mean** **(range) months**
Akeboshi *et al* (2004)	54/31	13/41	2.7 (0.7–6)	32/54 (59%)	9.2 (5–18)
Gadaleta *et al* (2004)	69/34	6/28	<10	58/63 (92%)	9 (3–25)
King *et al* (2004)	45/20	0/20 (colorectal)	<3.5	20/25 (80%) at 12 months	24 (5–31)
Lee *et al* (2004)	32/30	26/4	5.2 (1–12)	12/32 (38%)	12.5 (1–24)
Yasui *et al* (2004)	99/35	3/96	2.0 (0.3–8)	90/99 (91%)	7.1 (1–17)

**Table 2:** RFA in renal cell carcinoma

**Authors**	**No of tumours/** **patients**	**Tumour size mean** **(range) cm**	**Complete ablation –** **no of tumours**	**Length of follow-up** **mean (range) months**
Schiller *et al* (2005)	42/34	3.2 (1.1–8.9)	36/42 (86%)	9.9 (3–43)
Farrell *et al* (2003)	35/20	1.7 (0.9–3.6)	35/35 (100%)	9 (1–23)
Mayo-Smith *et al* (2003)	?/32	2.4 (1–5)	31/32 (97%)	9 (1–36)
Su *et al* (2003)	35/29	2.2 (1–4)	33/35 (94%)	9 (0–23)
